# RETRACTED: Dasari et al. Neferine Targets the Oncogenic Characteristics of Androgen-Dependent Prostate Cancer Cells via Inducing Reactive Oxygen Species. *Int. J. Mol. Sci.* 2023, *24*, 14242

**DOI:** 10.3390/ijms27010348

**Published:** 2025-12-29

**Authors:** Subramanyam Dasari, Nishtha Pathak, Amy Thomas, Shreeja Bitla, Raj Kumar, Gnanasekar Munirathinam

**Affiliations:** 1 School of Medicine, Indiana University Bloomington, Bloomington, IN 47405, USA; sudasari@iu.edu; 2 Department of Biomedical Sciences, University of Illinois College of Medicine, Rockford, IL 61108, USA; npatha4@uic.edu (N.P.); athom44@uic.edu (A.T.); sbitla3@uic.edu (S.B.); 3 Department of Biotechnology and Bioinformatics, Jaypee University of Information Technology, Waknaghat, Solan 173234, Himachal Pradesh, India; raj.kumar@juitsolan.in

The journal retracts the article titled “Neferine Targets the Oncogenic Characteristics of Androgen-Dependent Prostate Cancer Cells via Inducing Reactive Oxygen Species” [1], cited above.

Following publication, concerns were brought to the attention of the Editorial Office regarding image duplication within this article [1].

Adhering to our standard procedure, an investigation was conducted by the Editorial Office and Editorial Board that identified indications of inappropriate editing and partial duplication between the panels in Figure 3A in this article [1] presenting different experimental conditions. While the authors cooperated with the Editorial Office during the investigation, they were unable to satisfactorily explain the overlap nor provide raw material of an acceptable standard for Editorial Board evaluation. Consequently, the Editorial Board has lost confidence in the reliability of the findings and has decided to retract this publication [1], as per MDPI’s retraction policy (https://www.mdpi.com/ethics#_bookmark30).

This retraction was approved by the Editor-in-Chief of the *International Journal of Molecular Sciences*.

The authors did not provide a comment on this decision.
